# The Mississippi Valley Association

**Published:** 1863-04

**Authors:** 


### THE MISSISSIPPI VALLEY ASSOCIATION.

The Mississippi river hasn't turned its course up stream; and though it has been somewhat variously tapped, and miscellaneously blockaded, it pretty much glides along towards the Gulf. So the *Mississippi Association* hasn't retrograded; and though *tapped* by many local societies, and blockaded by the "war power," so as to lose one meeting, it goes along, carrying the commerce of professional thought on its tide. We, unfortunately, were not aboard during the last voyage. (It seems odd to have a meeting of the *old Society*, and our name not in the list of "members present.")

We are happy to learn that the Association has lost none of its energy—that the last meeting was a most profitable one to those present. We hope to have our brain connected with it by a "Lake Providence Canal" before the next meeting, that we may be flooded with new ideas and fresh resolves. W.

